# CT Angiography for Aortic Arch Anomalies: Prevalence, Diagnostic Efficacy, and Illustrative Findings

**DOI:** 10.3390/diagnostics14171851

**Published:** 2024-08-24

**Authors:** Radu Octavian Baz, Deria Refi, Cristian Scheau, Any Axelerad, Radu Andrei Baz, Cosmin Niscoveanu

**Affiliations:** 1 Clinical Laboratory of Radiology and Medical Imaging, “Sf. Apostol Andrei” County Emergency Hospital, 900591 Constanta, Romania; 2 Department of Radiology and Medical Imaging, Faculty of Medicine, “Ovidius” University, 900527 Constanta, Romania; 3 Department of Physiology, The “Carol Davila” University of Medicine and Pharmacy, 050474 Bucharest, Romania; 4 Department of Radiology and Medical Imaging, “Foisor” Clinical Hospital of Orthopaedics, Traumatology and Osteoarticular TB, 021382 Bucharest, Romania; 5 Department of Neurology, Faculty of Medicine, “Ovidius” University, 900527 Constanta, Romania

**Keywords:** aortic arch anomalies, right aortic arch, coarctation of aorta, pseudocoarctation, ductus diverticulum, aberrant subclavian artery, vascular anatomical variants, computed tomography, imaging

## Abstract

Aortic arch anomalies encompass a diverse spectrum of conditions. Elucidating the prevalence of these anomalies, their impact on patient wellbeing, and the most effective diagnostic tools are crucial steps in ensuring optimal patient care. This paper aims to explore the various presentations of aortic arch anomalies, emphasizing the remarkable utility of computed tomography (CT) angiography in their definitive diagnosis and characterization. We conducted a retrospective study on patients who were submitted to the CT angiography of the thoracic aorta or supra-aortic trunks, or the contrast-enhanced CT scans of the thorax and/or cervical region between January 2021 and February 2024 in our Hospital. Out of the total of 2350 patients, 18 were diagnosed with aortic arch anomalies, with an average age of approximately 55 years. The aortic arch anomalies identified in the study were as follows: left aortic arch with the aberrant origin of the right subclavian artery, right aortic arch (types I and II), double aortic arch, aortic coarctation, aortic pseudocoarctation, and ductus diverticulum. Although often asymptomatic, aortic arch anomalies require recognition and CT using advanced post-processing techniques is the optimal diagnostic method with the ability to also identify other associated cardiac or vascular malformations.

## 1. Introduction

Aortic arch anomalies, frequently presenting as asymptomatic entities, encompass a diverse array of anatomical variants that can profoundly impact a patient’s quality of life. These anomalies include abnormal emergence, position, trajectory, contour, or caliber of the arch and its branches [1,2,3].

The prevalence of aortic arch anomalies varies in the general population, with some types being more common than others [4,5]. The anomalies can affect patients of all ages; however, clinically significant anomalies will be diagnosed sooner, as they will have a more significant impact on the patient’s wellbeing [6].

Vascular variants and anomalies are of high clinical relevance due to their complex anatomy, potential for life-threatening complications, and surgical challenges [7,8,9,10,11]. Moreover, their sometimes-asymptomatic nature can delay the diagnosis and can lead to increased economic burden [12,13,14]. Patients may experience symptoms such as dyspnea, chest pain, or neurological deficits. In severe cases, these anomalies can lead to complications including aortic dissection, aneurysm formation, or the compression of adjacent structures [15,16,17].

Traditional imaging modalities often pose diagnostic challenges due to insufficient detail for accurate assessment [18,19]. While echocardiography primarily serves to evaluate cardiac function, it can also effectively delineate the aortic arch’s course and branching pattern, providing valuable information for planning subsequent cross-sectional imaging studies [20].

Computed tomography (CT) with CT angiography (CTA) is essential in the diagnosis and evaluation of aortic arch anomalies and associated features and has numerous advantages such as short acquisition time, increased spatial resolution, and good anatomic coverage [21,22,23].

Magnetic resonance angiography (MRA) may represent a diagnostic alternative; however, it has long examination times, may require sedation in uncompliant children, and the data provided regarding the compression effect on surrounding structures are scarce [24,25]. Nonetheless, advancements like 3D volumetric acquisition, 3D ultra-shot echo, and 4D flow acquisition techniques are emerging as promising future solutions to these challenges [21]. MRI is particularly useful in patients with contraindications to CT examinations, or when a detailed assessment of the perivascular soft tissues is needed. Dedicated vascular applications have long been applied to assess configuration variations for different vascular territories [26].

Cardiac MRI (cMRI) is a non-invasive and radiation-free imaging technique that excels in visualizing heart function, blood flow, and tissue structure, making it valuable for diagnosis and treatment planning. While not always the initial choice, cMRI is essential for complex cases, especially in children and post-surgery evaluation. It aids in detecting and assessing heart defects, planning interventions, and monitoring treatment outcomes [27].

Adequate equipment, methods, and technical parameters are required in order to obtain a high-quality and relevant investigation [28,29]. Image post-processing is often required to better visualize adjacent anatomical structures and, when appropriate, to correctly set up the surgical planning [30,31].

This article is a comprehensive review with a case series which aims to assess the incidence and types of aortic arch anomalies and explores a selection of the numerous congenital defects; our objectives were to emphasize the role of CTA in deciphering the complexity of aortic arch variations and to thoroughly describe and illustrate them. By facilitating accurate diagnosis, surgical planning (when necessary), and the monitoring of potential complications or treatment response, CT serves as a cornerstone in managing these anomalies.

## 2. Embryology and Anatomy

For a better understanding of the development of the great vessels, a brief review of their embryology is necessary. The most popular theories of their formation are Rathke’s diagram and Edward’s double arch model. The development of the great vessels begins around the 3rd gestational week and is largely complete by the 8th week [32].

Rathke’s diagram proposes that the great vessels originate from the six pairs of branchial (pharyngeal) arches. Each arch connects the primitive ventral aortas (which will fuse to create the aortic sac) with the two dorsal aortas. These primitive arches appear successively, in a cranio-caudal order, regress, remodel, and ultimately give rise to the system of great vessels, including the aortic arch, supra-aortic trunks, the ductus arteriosus, and the proximal segments of the pulmonary arteries [33].

The first two primitive arches will regress, with their remaining portions becoming the maxillary, hyoid, and stapedial arteries. The segments located between the third and fourth arches of the ventral aortas will develop into the common carotid arteries, while the third pair of pharyngeal arches, together with the overlying portions of the dorsal aortas, will give rise to the internal carotid arteries. The external carotid arteries form as new branches of the third pair of arches [34]. The sixth arch on the left side forms the pulmonary trunk, left pulmonary artery, and ductus arteriosus, while the one on the right contributes to the development of the right pulmonary artery [35].

The segment of the left ventral aorta between the fourth and sixth arches, along with the left fourth intersegmental artery and the dorsal aorta on the same side, form the aortic arch. The brachiocephalic trunk (originating from the portion of the right ventral aorta between the fourth and sixth arches), the left common carotid artery, and the left subclavian artery (developed from the seventh intersegmental artery) branch off from the aortic arch [34,36]. The fourth and sixth arches on the right side form the right subclavian artery [36].

In contrast to the previous model, Edward’s double aortic arch model proposes the existence of a single ascending and descending aorta located in the midline, along with an aortic arch and ductus arteriosus on each side of these structures, surrounding both the trachea and the esophagus. The carotid and subclavian arteries are each formed from the ipsilateral arch. Specific interruptions of this system will lead to variants of the aortic arch. The normal aortic arch arises from the interruption of the dorsal segment of the right aortic arch, at the level between the right subclavian artery and the descending aorta, while the ductus arteriosus regresses [37].

The aortic arch considered normal in humans is located to the left of the midline and from its convexity arise, from right to left, the three supra-aortic trunks: the right brachiocephalic arterial trunk (subsequently giving rise to the common carotid and subclavian arteries), the left common carotid artery, and the left subclavian artery. The ligamentum arteriosum (also known as Botallo’s ligament) stretches between the left pulmonary artery and the aortic portion located distal to the emergence of the left subclavian artery [33,38].

Essentially, Rathke’s diagram is based on the existence of two separate dorsal aortas connected by multiple branchial arches, while Edwards’ model proposes a midline aorta with paired aortic arches and ductus arteriosus encircling the trachea and esophagus [37,39].

As key differences, Rathke’s diagram emphasizes an asymmetrical development of the aortic arches, resulting in a wider range of aortic arch anomalies due to its complex structure, while Edwards’ model focuses on a symmetrical arrangement, primarily explaining the formation of a double aortic arch.

Understanding the normal development of the aortic arch is crucial for comprehending the pathogenesis of aortic arch anomalies. Deviations from this normal sequence can result in various abnormalities:

- Abnormal persistence: the continued presence of embryonic structures that should normally regress, such as the fourth aortic arch on both sides, can lead to a double aortic arch as described in Edwards’ model [40].
- Abnormal regression: the incomplete development or disappearance of the right dorsal aorta in the portion between the right common carotid artery and the right subclavian artery leads to an aberrant origin of the latter [37], a common anatomical variation. This anomaly can have significant clinical consequences, including altered positioning of the recurrent laryngeal nerve, thereby increasing the risk of nerve damage during thoracic surgery. Additionally, it can contribute to severe bleeding during esophagectomy or esophageal dissection [41].
- Aberrant connections: abnormal connections between the aortic arches and other vessels can lead to complex aortic arch anomalies, such as the right aortic arch with an aberrant subclavian artery [37].

By correlating the embryological development of the aortic arch with the clinical presentation of its anomalies, clinicians can better grasp the underlying mechanisms and devise effective diagnostic and treatment plans.

## 3. Materials and Methods

A retrospective study was conducted on 2350 patients aged 25 to 91 years old, examined between January 2021 and February 2024 in the Clinical Radiology and Medical Imaging Laboratory of County Emergency Clinical Hospital “Sf. Apostol Andrei” Constanta. The inclusion criteria were as follows: patients who underwent CTA of the thoracic aorta or cervical arteries or CT scans with contrast medium of the thoracic and/or cervical region. The exclusion criteria were patients with surgeries, stents, or other procedures on the aorta; pregnancy; renal failure; a history of allergic reactions to iodinated contrast medium; and patients that did not consent for their data to be used in medical research. Aortic arch anomalies were identified using the radiology reports stored in the hospital registry and all the identified cases (*n* = 18) were reviewed and thoroughly analyzed. All the images were stored in the hospital image database. None of the identified cases of aortic arch anomalies were excluded due to incomplete or missing data. The cases were classified in a subtype of aortic arch anomaly based on a consensus of at least 2 senior radiologists in order to mitigate any reviewer bias. As our study was designed only to assess the prevalence of these variations and to describe the CTA morphology, no additional statistical analysis was performed.

The indications for the examinations were varied, including digestive, pulmonary, or cardiac manifestations (among the most common symptoms were dysphagia, dysphonia, dyspnea, angina pectoris, and palpitations).

The examinations were conducted using the Revolution Apex™ 512-detector CT scanner (General Electric, Fairfield, CT, USA). The patients were placed in a dorsal decubitus position and instructed to maintain inspiratory apnea during image acquisition. With the help of a dual injector, a quantity of iodinated contrast medium varying between 60 and 100 mL (1.5 mL/kg body weight) was administered intravenously at a speed between 2 and 4.5 mL per second, followed by 20–30 mL of saline solution at the same injection rate. Post-contrast scanning was performed, depending on the protocol required, as follows:

- For angiographic investigations, the acquisition consisted of thin sections with a thickness of 0.625 mm;
- For routine CT examinations, the slice interval in the initial acquisition was 5 mm, with thinner reconstructions of 1.25 mm or 2.5 mm.

Subsequently, the axial images obtained were transferred to a workstation that allowed post-processing and the obtaining of images through maximum intensity projection (MIP), multiplanar reformatting (MPR), curved multiplanar reformatting (cMPR), and volume rendering technique (VRT).

## 4. Results

Of the 2350 cases, 18 patients (0.77%) aged between 32 and 84 years (average age 55.2 years) presented with aortic arch anomalies. These patients included 7 females (38.89%) and 11 males (61.11%). The distribution of aortic arch anomalies was as follows: eight cases (44.44%) with left aortic arch and the aberrant origin of the right subclavian artery, four cases (22.22%) with right aortic arch (1 type I and 3 type II), two cases (11.11%) of ductus diverticulum, and one case each (5.55%) of double aortic arch, coarctation, pseudocoarctation of the aorta, and patent ductus arteriosus (summarized in Table 1).

Among the cases with the aberrant origin of the right subclavian artery, seven emerged from the medial wall of the aortic arch. One case originated from the posterior wall of the aortic arch and was associated with the congenitally corrected transposition of great vessels and the abnormal origin of the right vertebral artery from the ipsilateral common carotid artery.

An illustrative case series is further included to demonstrate the characteristics of the aortic arch anomalies, depict the CT angiography features of each subtype and associated findings, as well as to assess the potential clinical impact.

### Case Series

**Figure 1:**
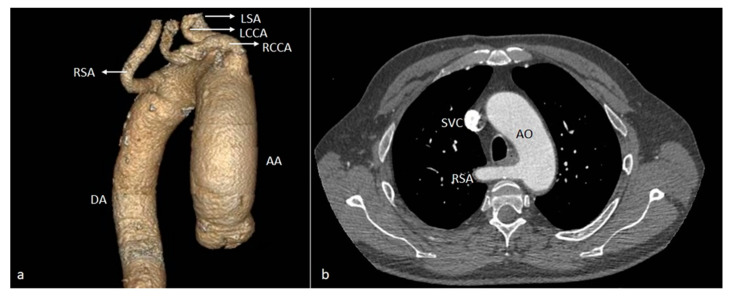
VR (**a**) and axial (**b**) CT images showing the aberrant origin of the right subclavian artery from the medial wall of the aortic arch with a retroesophageal course. AA—ascending aorta. DA—descending aorta. RCCA—right common carotid artery. LCCA—left common carotid artery. RSA—right subclavian artery. LSA—left subclavian artery. SVC—superior vena cava. AO—aorta.

**Figure 2:**
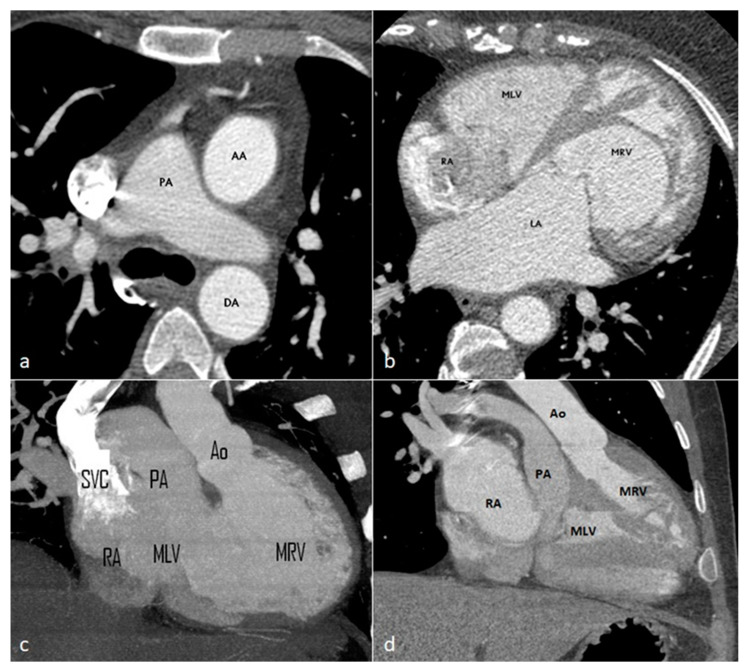
Axial (**a**,**b**) and coronal (**c**,**d**) contrast-enhanced CT images showing the levotransposition of the aorta (the ascending aorta located anterior and to the left of the main pulmonary artery) and four chambers of the heart, with the left upper cardiac border formed by the inverted aorta and anatomic right ventricular outflow tract. PA = pulmonary artery. AA = ascending aorta. DA = descending aorta. LA = left atrium. RA= right atrium. MRV = morphologic right ventricle. MLV = morphologic left ventricle. Ao = aorta. SVC = superior vena cava.

**Figure 3:**
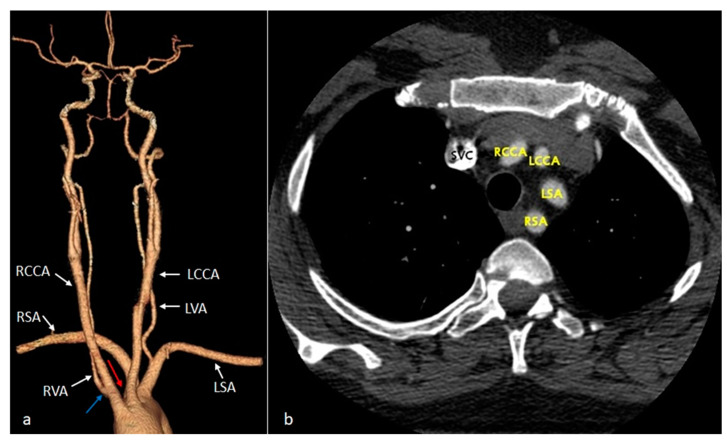
VR (**a**) and axial (**b**) CT images showing the aortic arch with the absence of the brachiocephalic trunk and the direct emergence from this level of the four arterial branches. Note the origin of the RSA from the posterior wall of the aortic arch (red arrow), and the RVA from the RCCA (blue arrow). RCCA—right common carotid artery. LCCA—left common carotid artery. LSA—left subclavian artery. RSA—right subclavian artery. SVC—superior vena cava. LVA—left vertebral artery. RVA—right vertebral artery.

*Aberrant (anomalous) origin of the right subclavian artery (ARSA)* consists of the distinct emergence of this artery from the distal part of the aortic arch, as the last of the supra-aortic trunks, carrying a course behind the esophagus towards the right axillary region (Figure 1). In our patient cohort, it was by far the most prevalent variant, seen in eight cases (44.44% of the total patients), all of whom had a retroesophageal course. Among these, one case was associated with the congenitally corrected transposition of great vessels—the only cardiac malformation encountered in the study (Figure 2)—and the abnormal origin of the right vertebral artery, emerging from the ipsilateral common carotid artery (Figure 3).

**Figure 4:**
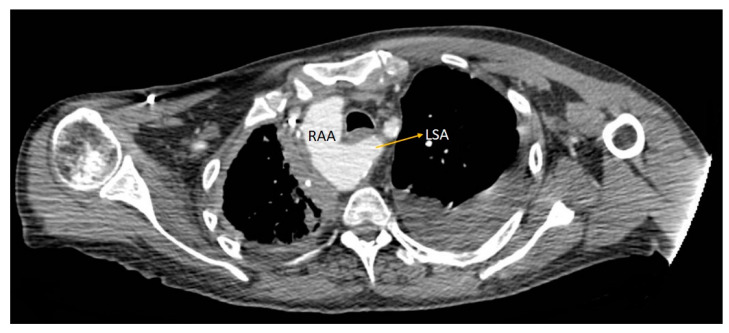
Axial CT image showing type II right aortic arch, with the aberrant origin of the left subclavian artery, emerging from the medial wall of the aortic arch, following a retroesophageal course (orange arrow). RAA—right aortic arch. LSA—left subclavian artery.

**Figure 5:**
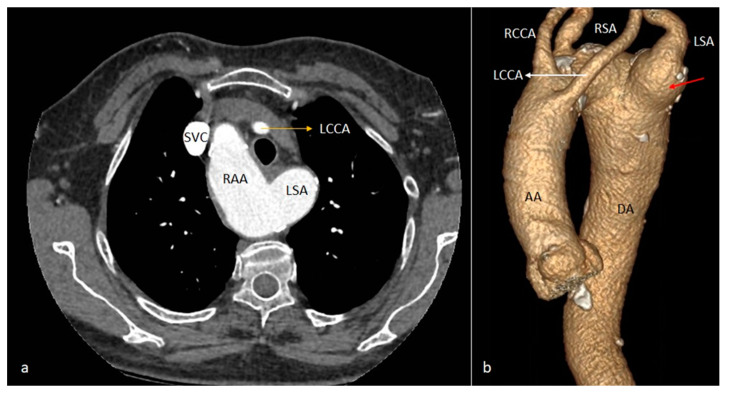
Axial CT (**a**) and VR (**b**) images showing a type II right aortic arch (RAA) and the aberrant origin of the left subclavian artery (LSA). Kommerell’s diverticulum shows as a focal ectasia at the base of the LSA origin (red arrow). SVC—superior vena cava. RAA—right aortic arch. LCCA—left common carotid artery. LSA—left subclavian artery. RCCA—right common carotid artery. RSA—right subclavian artery. AA—ascending aorta. DA—descending aorta.

Four cases (22.22%) of *right aortic arch (RAA)* were identified. Of these, three (17.64%) were represented by type II RAA (the most common type in the general population), in which the first branch emerging from the aortic arch is the left common carotid artery, followed by the right common carotid artery, right subclavian artery, and aberrantly originating left subclavian artery (Figure 4). In one of the patients in our study, the anomalous origin of the left subclavian artery arose from the persistence of the left dorsal segment of the aortic arch, a dilated area at the emergence known as Kommerell’s diverticulum (Figure 5).

**Figure 6:**
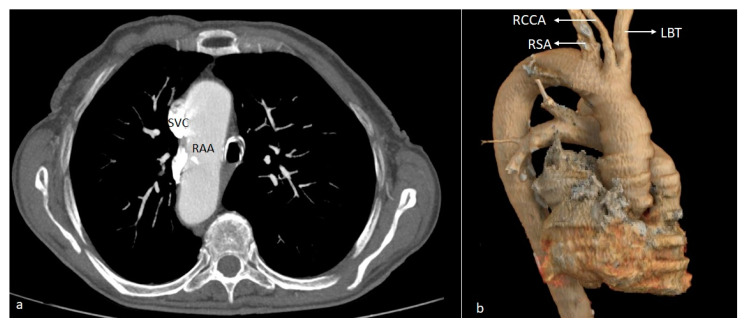
Axial CT (**a**) and VR (**b**) images illustrating a type I right aortic arch (RAA) with “mirror image” emergence of the supra-aortic trunks. SVC—superior vena cava. RAA—right aortic arch. LBT—left brachiocephalic trunk. RCCA—right common carotid artery. RSA—right subclavian artery.

In one case (5.55%) of right aortic arch (RAA), subtype I was identified with a “mirror image” emergence of the supra-aortic trunks, a rarer variant. The brachiocephalic trunk is the first to emerge from the aortic arch, followed by the right common carotid and right subclavian arteries (Figure 6).

*Double aortic arch (DAA)* is the most often encountered type (42%) in a group of defects which are entitled “vascular rings”. [42]. It refers to a complete surrounding and potential compression of the trachea and/or esophagus by the DAA, by its branches, or, rarely, by hypoplastic ligamentary segments. Most frequently, the RAA is dominant and is situated cranially to the left one [42].

**Figure 7:**
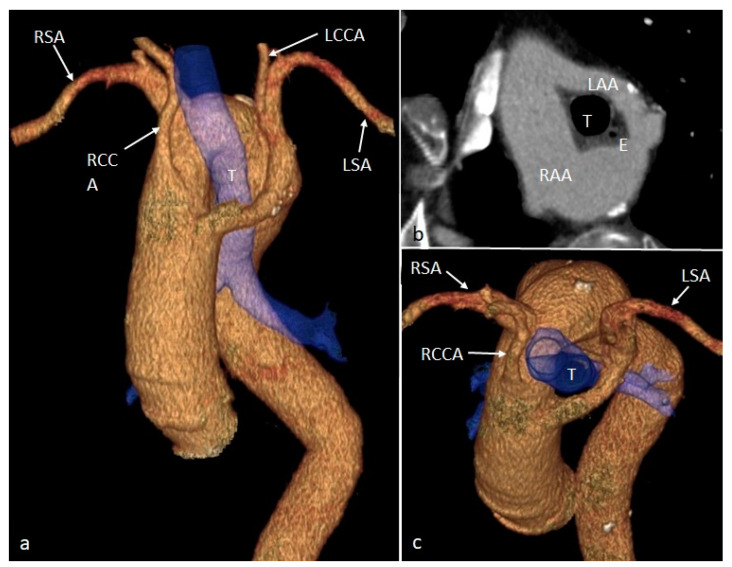
VR (**a**,**c**) and oblique CT (**b**) images showing the double aortic arch (with right dominance—RAA), encircling the trachea (T) and esophagus (E), with a significant stenotic effect on the latter. Each aortic hemiarch (right and left) gives rise to the common carotid and subclavian arteries of the same side. RSA—right subclavian artery. RCCA—right common carotid artery. LCCA—left common carotid artery. LSA—left subclavian artery. RAA—right aortic arch. LAA—left aortic arch. T—trachea. E—esophagus.

The case identified in our patient cohort was an adult patient with dysphagia. CT imaging of the thorax revealed a double aortic arch (with right dominance) that caused a significant extrinsic compressive effect on the trachea and, especially, the esophagus (Figure 7). The right and left segments each independently supply the corresponding common carotid and subclavian arteries.

**Figure 8:**
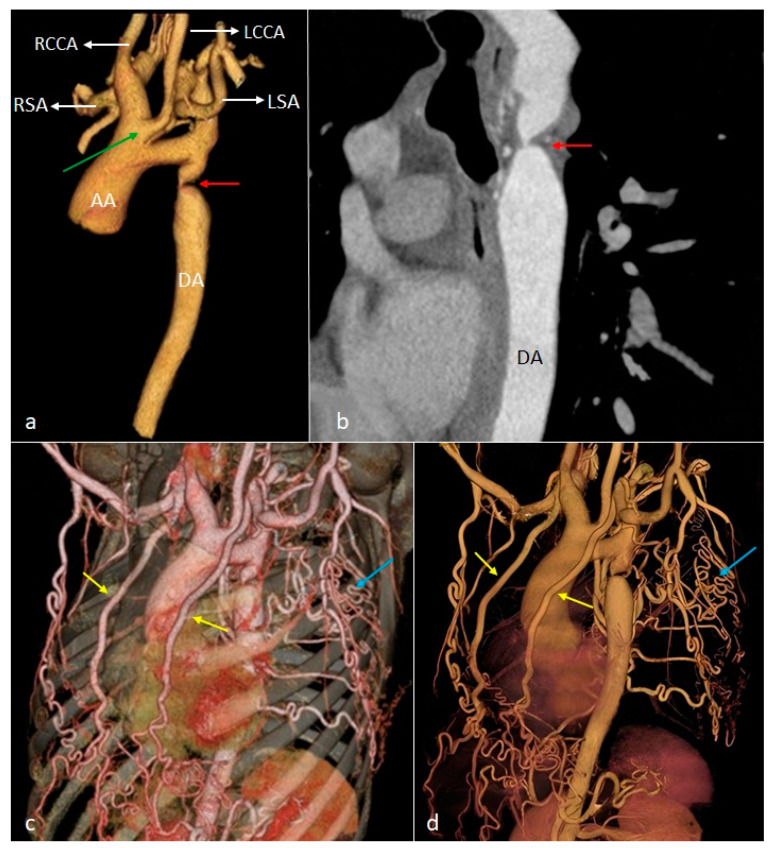
VR (**a**,**c**,**d**) and cMPR (**b**) images illustrating aortic coarctation, visible as the stenosis of the aortic arch distal to the emergence of the left subclavian artery and below the insertion of the arterial ligament (red arrows), associated with dilation of the internal mammary arteries (yellow arrows) and intercostal arteries (blue arrows). Incidentally, the common origin of the brachiocephalic trunk and left common carotid artery is noted (green arrow). AA—ascending aorta. DA—descending aorta. RCCA—right common carotid artery. LCCA—left common carotid artery. RSA—right subclavian artery. LSA—left subclavian artery.

*Aortic coarctation* represents a focal stenosis of the aortic arch lumen and can be preductal (infantile) or postductal (adult) [43]. Our only case (5.55%) was of the postductal type (Figure 8), found in a middle-aged patient.

**Figure 9:**
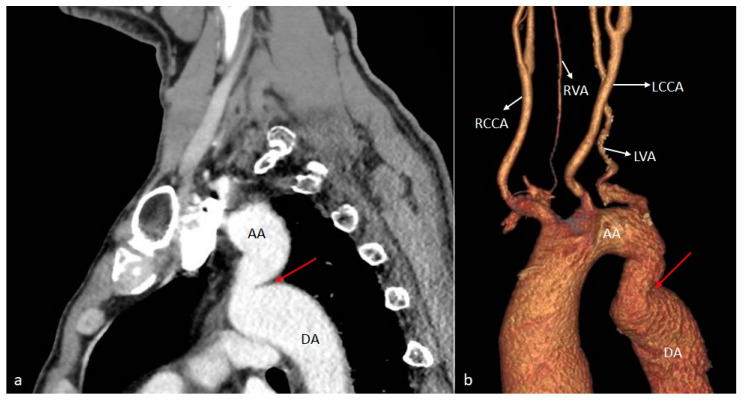
MPR sagittal (**a**) and VR (**b**) CT images show a periductal narrowing and kinking of the aorta (red arrows) without the subsequent dilation of the supra-aortic trunks, intercostal and internal mammary arteries, findings suggestive of the pseudocoarctation of the aorta. AA—aortic arch. DA—descending aorta. RCCA—right common carotid artery. LCCA—left common carotid artery. LSA—left subclavian artery. RVA—right vertebral artery. LVA—left vertebral artery.

*Pseudocoarctation of the aorta* is a rare anomaly of the aortic arch, characterized by a kinking of the aorta at the level of the isthmus. The case identified in our cohort presented a mild kink, with an elongated aortic arch, no significant associated narrowing of the aortic lumen, and no visible collateral circulation pathways (Figure 9).

**Figure 10:**
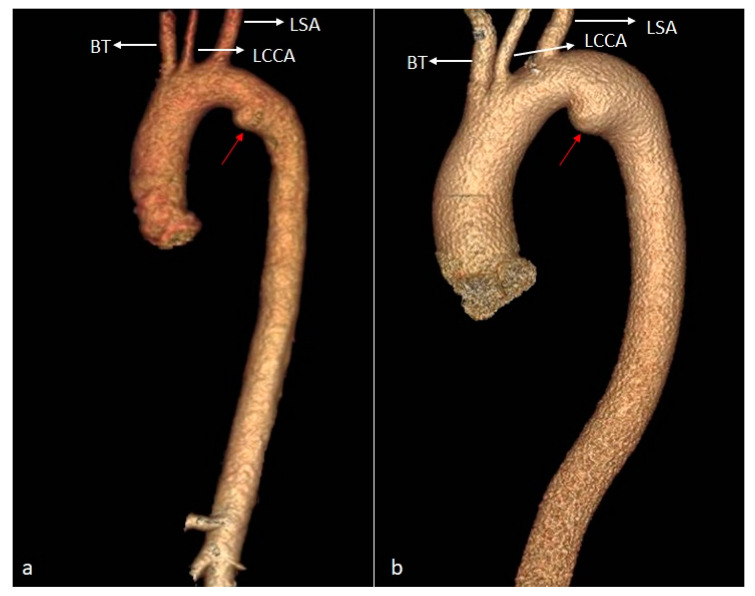
VR images ((**a**)—case 1, (**b**)—case 2) showing ductus diverticulum—a smooth focal bulge forming obtuse angles with the aortic wall, seen usually in the anteromedial aspect of the aortic isthmus, where the ligamentum arteriosum attaches (red arrows). BT—brachiocephalic trunk. LCCA—left common carotid artery. LSA—left subclavian artery.

Out of the two cases of *ductus diverticulum*, one was discovered incidentally in a young female patient who underwent an upper extremity CT angiogram to evaluate the patency of a brachiocephalic arteriovenous fistula. The other case was that of a male patient with arterial hypertension, referred for CT angiography for a suspicion of ascending aorta ectasia. In both cases, the CT scan revealed small saccular dilatations at the level of the aortic isthmus, in the projection area of the former aortic insertion of the ductus arteriosus (Figure 10).

**Figure 11:**
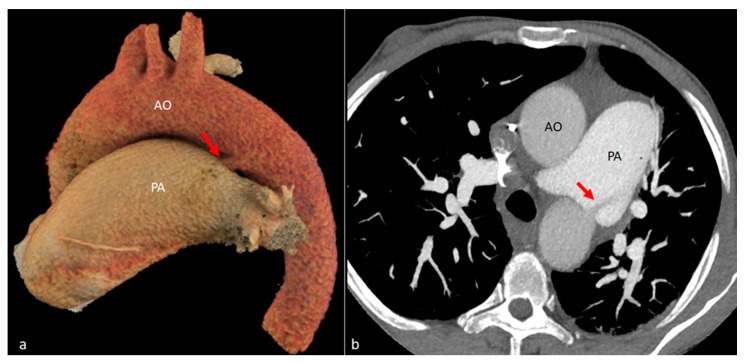
VR (**a**) and MIP (**b**) images show a tubular structure connecting the pulmonary artery and the distal segment of the aortic arch, with unenhanced blood seen flowing from the aorta to the pulmonary artery via patent ductus arteriosus (red arrows). Note the markedly enlarged caliber of the pulmonary artery. AO = aorta. PA = pulmonary artery.

While not necessarily a type of aortic arch anomaly, we decided to include the case of a 51-year-old male with *known patent ductus arteriosus* (Figure 11), who underwent a CT scan of the thoraco-pulmonary region for preoperative assessment. Patent ductus arteriosus can be sometimes mistaken for a persistent fifth aortic arch which occurs due to lacking of physiologic regression. The remnant arch can connect the ascending segment of the aortic arch to the descending aorta (systemic–systemic) or pulmonary artery (systemic–pulmonary). Due to its location and connection, the persistent fifth aortic arch can mimic a persistent ductus arteriosus.

## 5. Discussion

Anatomical variants of the aortic arch are found with a variable frequency in the literature, estimated at approximately 1–2% of the general population [44], but this is dependent on the specific subtype of the anomaly and the diagnostic methods used. In our study, the prevalence was 0.77%, similar to the results obtained in another study in the literature [45].

Aortic arch anomalies were divided by Bae et al. into three broad categories: left aortic arch, right aortic arch, and other aortic arch variants [21]. Among these, the left or right aortic arches may associate aberrant or isolated subclavian artery or circumflex aorta, while other variants include double aortic arch, cervical, interrupted, hypoplastic or persistent fifth aortic arch, and also coarctation and the pseudocoarctation of the aorta [21].

The most common anomaly of the aortic arch is the left aortic arch with the aberrant origin of the right subclavian artery, found in approximately 0.4–2% of the general population [46], and was also the predominant type found in our study, in almost half of the cases (44.4%).

The cause of the aberrant origin of the right subclavian artery is the regression of the right dorsal aorta in the portion between the right common carotid artery and the right subclavian artery [37], usually identified as an isolated anomaly.

Depending on its relationship to the esophagus, the course of the artery can be retroesophageal (the most common type, also seen in all the cases found in our patients), in between the esophagus and trachea, or pretracheal [47]. Although very rarely symptomatic, most often discovered incidentally, in the event that it manifests with dyspnea or dysphagia (“dysphagia lusoria”—mainly seen in children) surgical treatment may be considered [21]. However, in patients who require surgery of the upper mediastinum (e.g., esophagectomy for esophageal cancer), even asymptomatic variants of this anomaly pose difficulties, presenting a risk of arterial injury [48].

A special case was individualized by the association of the aberrant origin of the right subclavian artery with the abnormal origin of the right vertebral artery from the ipsilateral common carotid artery in a patient known with congenital corrected transposition of great vessels. Similar to a case described in a cadaveric study by Roszel and Kiely [49], an aortic arch with separate emergence of four supra-aortic branches was also detected in our patient: the first branches to emerge from the convexity of the aortic arch, at a short distance from each other, were the right and left common carotid arteries, followed by the left subclavian artery and, finally, the right subclavian artery. The latter, emerging from the posterosuperior aspect of the arch, subsequently followed a retroesophageal ascending course to the right upper limb. In association, the origin of the right vertebral artery from the ipsilateral common carotid artery was observed.

As recognized in the literature, transposition of the great vessels is frequently associated with ventricular septal defect, obstruction of the left ventricular outflow tract, and aortic arch anomalies [50], the latter identified in approximately 14% of the cases [51].

Regarding the aberrant origin of the right subclavian artery associated with transposition of great vessels, according to a study by Zapata et al., it is only found in 2% of the cases, more frequently observed in the context of other congenital malformations such as tetralogy of Fallot, double-outlet right ventricle or persistence of the ductus arteriosus [52].

Right aortic arch has an incidence of approximately 0.05% in the general population [53], and was the second most common anomaly found (22.2%) in our patient cohort. Depending on the mode of origin of its branches, it is classified into three subtypes: with “mirror” branching of the supra-aortic trunks (type I), with the aberrant origin of the left subclavian artery (type II), and with the isolated left subclavian artery (type III).

A persistent right fourth branchial arch is the underlying cause of an abnormal right aortic arch. The right dorsal aorta persists, becoming the thoracic aorta, while the left dorsal aorta regresses [54].

Type II is the most frequent [53], being caused by an interruption of the dorsal segment of the left arch between the origin of the common carotids and left subclavian arteries, with regression of the ductus arteriosus [37]. In this type, the first branch is the LCCA, followed by the RCCA, RSA, and LSA. The persistence of the dorsal segment of the sixth pharyngeal arch on the left side leads to the formation of a retroesophageal pouch, also known as Kommerell diverticulum. This diverticulum subsequently gives rise to the left subclavian artery. It is rarely symptomatic, but patients with aneurysmal Kommerell diverticulum are at increased risk of esophageal compression or spontaneous rupture, which is why they are candidates for surgical treatment [55].

Other possible manifestations of type II right aortic arch are the early onset of atherosclerotic changes, the risk of dissection or aneurysmal dilation, and the compressive effect on adjacent structures [56]. Atherosclerotic deposits in the collateral circulation branches can increase retrograde flow to the left vertebral artery, giving rise to a subclavian steal syndrome, associated with an increased risk of stroke [57]. The reconstruction of the left subclavian artery can prevent complications such as upper limb claudication or subclavian steal syndrome, which can represent prognostic factors, particularly in the context of endovascular interventions [44,56].

Type I right aortic arch, with “mirror” emergence of the supra-aortic trunks, ranks second in frequency, with an incidence in the general population of between 0.012 and 0.018% [58]. As its name suggests, it is a mirror image of the normal left aortic arch, the order of detachment of its branches being as follows: the (left) brachiocephalic arterial trunk, followed by the right common carotid and right subclavian arteries. This variant results from the regression of the left dorsal aorta distal to the origin of the seventh intersegmental artery so that the left fourth branchial arch will form the portion of the ipsilateral subclavian artery, instead of contributing to the formation of the aortic arch [59]. Similar to type II right aortic arch, this variant also predisposes to the association with early atherosclerotic disease, and aneurysmal dilatations [59], its clinical importance being given by the mortality associated with aneurysmal ruptures, as well as by the fact that in over 75% of the cases it is accompanied by other anomalies or congenital heart malformations [60,61].

Type III right aortic arch with an isolated left subclavian artery is the rarest form, in which the left subclavian artery is connected either to the pulmonary artery (via the ductus arteriosus) or to the vertebral artery on the same side [62] and is the result of the regression of two segments of the left aortic arch—both the one between the common carotid and subclavian arteries on the left side, as well as the one located distal to the ductus arteriosus [46]. If the ductus arteriosus is obliterated, retrograde blood flow to the left vertebral artery or collateral circulation pathways occurs [60] and as seen in the first two types of right aortic arch, there is a subclavian vascular steal syndrome, vertebrobasilar insufficiency, and the challenge of endovascular interventions [63].

The double aortic arch is a rare but well-documented congenital vascular anomaly, representing the most common form of “vascular ring” malformation (42% of the total) and is responsible for 1% of all congenital heart defects [64]. The presence of a vascular ring that surrounds the trachea and esophagus can manifest through a variety of symptoms, depending on the severity of compression exerted on these structures. Manifestations at a young age include dyspnea, stridor, dysphagia, feeding problems, and recurrent respiratory infections [65,66]. Alternatively, similar to our patient, adult presentations can occur with chronic swallowing difficulties, or some individuals may remain asymptomatic when tracheo-esophageal compression is minimal. In these cases, diagnosis is often incidental during investigations for unrelated conditions [67,68,69].

In normal embryonic development, during the fifth week of gestation, the right fourth aortic arch undergoes regression, leaving behind a blood vessel that eventually forms the typical left aortic arch. The persistence of the fourth pair of aortic arches, both right and left, produces a vascular configuration with a double aortic arch [40].

The two aortic arches can be of a similar caliber or one of them can be atretic or hypoplastic. The three subtypes of the double aortic arch are classified according to the relative size of the right and left segments of the double aortic arch and their location in relation to the trachea: dominant right aortic arch (which occurs in 75% of the cases), dominant left aortic arch (20% of the cases) and balanced aortic arches (5% of the cases) [70]. In our patient’s case, the right aortic arch had a larger diameter compared to the left arch. Additionally, the right arch was positioned cranially and posteriorly in the chest cavity compared to the left arch. The left arch, in contrast, appeared hypoplastic. This specific presentation, with a dominant right aortic arch and a smaller left arch, is the most common way this anomaly appears in patients.

Aortic coarctation, a focal narrowing of a specific aortic segment, can be located either before or after the ductus arteriosus and contributes to roughly 5–7% of all congenital heart malformations [71,72], with varying degrees of aortic arch or aortic isthmus hypoplasia.

The exact embryonic mechanism leading to the formation of aortic coarctation is not yet fully understood, but three hypotheses have been proposed in the literature. The first of these involves the presence of ductal tissue that extends to the aortic isthmus and, after postnatal closure of the ductus arteriosus, produces aortic obstruction and becomes a predisposing factor for coarctation [73]. Another theory includes hemodynamic alterations, where an abnormal preductal blood flow or an unnatural angle between the ductus arteriosus and the aorta will lead to decreased blood flow to the aortic arch and isthmus, resulting in the formation of aortic coarctation [74]. The last hypothesis implies an abnormal regression of a small part of the left dorsal aorta, which can rise with the formation of LSA and lead to aortic coarctation [73].

From a hemodynamic standpoint, a pressure gradient exceeding 20 mm Hg strongly suggests aortic coarctation [75]. In response to the obstruction of blood flow, the body initiates the development of collateral circulation pathways to maintain perfusion.

The most common malformations associated with aortic coarctation include bicuspid aortic valve and both ventricular and atrial septal defects [76]. Symptoms appear when there is a significant stenosis and collateral circulation is not effective. Patients with aortic coarctation are also predisposed to neurovascular complications such as intracranial hemorrhage, subarachnoid hemorrhage, and spinal hemorrhage [77,78,79].

Surgical correction of aortic coarctation is strongly recommended as early as possible, given the high mortality rate (up to 50%) and long-term sequelae associated with this condition [80,81]. However, it is important to note that even after successful surgical or endovascular correction, there is a relatively high risk of recurrent coarctation, aneurysm formation, and persistent primary hypertension [82].

The pseudocoarctation of the aorta is an uncommon congenital heart defect characterized by a thickening of the aortic wall at the isthmus. Unlike true aortic coarctation, pseudocoarctation does not cause hemodynamic obstruction or the development of collateral circulation networks [83].

One proposed embryological mechanism suggests that pseudocoarctation arises from the incomplete fusion of segments three to ten of the dorsal aortic roots and the left fourth branchial arch during the ascent of the seventh dorsal intersegmental artery [84].

Nearly all cases of pseudocoarctation are isolated and discovered incidentally in asymptomatic individuals. Surgical intervention is typically only necessary when pseudocoarctation is associated with an aortic aneurysm [85].

Despite its asymptomatic nature, some experts argue that pseudocoarctation should not be considered entirely benign. Its presence may contribute to hemodynamic conditions that favor the development of aneurysmal dilations, and therefore, dynamic monitoring is recommended in all cases [86].

Ductus diverticulum is a small outpouching at the site where the ductus arteriosus, a fetal blood vessel, normally closes after birth. It is considered a remnant of this structure and is found in approximately 9% of adults. Typically, this entity is asymptomatic and has no clinical significance. On CT scans, ductus diverticulum appears as a focal dilation at the aortic isthmus, most commonly located on the anteromedial wall, forming obtuse angles with the aortic wall [87]. In uncommon presentations, ductal diverticula may undergo aneurysmal transformation. However, surgical intervention is solely warranted when the diverticular diameter surpasses 3 cm. Precise identification of this anatomic variant holds significant importance for its differentiation from traumatic aortic pseudoaneurysms, which require immediate intervention due to their potential for rupture [88].

The ductus arteriosus originates from the posterior segment of the sixth aortic arch, completing its development by the eighth week of gestation [89]. In contrast, patent ductus arteriosus is characterized by the persistent patency of the ductus arteriosus beyond the expected timeframe of physiological closure, which typically occurs within 72 h after birth [90]. Although persistent ductus arteriosus is not traditionally classified as an aortic arch anomaly due to its categorization as a congenital cardiac malformation, it warrants discussion in this context due to its potential association with aortic arch anomalies [44,91,92]. Additionally, it can mimic the appearance of a ductal diverticulum, and a key differentiating feature lies in the absence of a connection to the left pulmonary artery in the case of a ductus diverticulum [93].

On CT images, the presence of a diminutive tubular structure, either independent from or connected to the aorta or pulmonary artery, raises suspicion for patent ductus arteriosus. Definitively diagnosing this entity requires confirmation of a continuous, patent connection between the aorta and the pulmonary artery demonstrating evident blood flow—the presence of a “flow void” signifying unenhanced blood coursing from the aorta to the pulmonary artery, or an “enhanced jet” of contrast-filled blood flowing from the aorta to the unenhanced pulmonary artery confirms the existence of a shunt [94]. CT facilitates both the quantitative and qualitative assessment of patent ductus arteriosus, at the same time allowing for a comprehensive evaluation of the surrounding structures, particularly helpful in identifying any coexisting abnormalities, potential complications, or assessing pulmonary hypertension signs, which might be relevant for overall patient management.

Identifying an aortic arch anomaly necessitates a thorough assessment of the cardiac anatomy. This is due to the well-established association between aortic arch anomalies and other congenital heart defects, such as ventricular septal defect, tetralogy of Fallot, truncus arteriosus, and transposition of the great arteries [65]. Furthermore, a link exists between aortic arch anomalies and chromosomal abnormalities like DiGeorge syndrome (22q11 deletion syndrome) [95]. Familiarity with the spectrum and imaging characteristics of aortic arch variations, anomalies, and malformations is essential for the accurate diagnosis, classification, and management of these conditions.

As previously mentioned, the adequate visualization and correct interpretation of aortic arch variants ensure that the medical team understands the anatomy and is capable of performing a competent and realistic intervention. Aortic arch anomalies are of particular interest in neuroradiology and cardiovascular surgery [63,96]. The complexity of the anatomy may be further complicated by the small vessel sizes of children [97]. It is recommended that the heart is also investigated in order to visualize potential associated coronary or cardiac variants or malformations [98,99]. Recent technical advancements allow for the use of 3D printing of the aortic arch and branches in order to aid preoperatory planning, orient the surgeon towards the required devices and instruments, or improve the overall learning process thus decreasing complications [100,101,102,103,104].

Our study acknowledges several limitations that affect how its findings can be interpreted. First, the study design was retrospective and conducted at a single center. Therefore, our data only reflects a specific patient population—those who underwent thoracic and/or cervical examinations at this particular center. Second, the study population leans heavily toward adults and older individuals. This is partly because CT scans, due to radiation exposure concerns, are typically reserved for these age groups. Also, asymptomatic individuals are more likely underrepresented as they are less likely to be submitted to diagnostic imaging. Additionally, symptomatic aortic arch variations may present early in life and be surgically corrected before adulthood, further contributing to this age bias. Therefore, due to these limitations, the prevalence rates reported in this study may not accurately reflect the true prevalence of aortic arch anomalies in the general population. The specific characteristics of the study population, such as the overrepresentation of older adults and the focus on individuals who underwent CT scans, limit the generalizability of the findings. Consequently, the conclusions drawn from this study should be interpreted with caution and considered applicable only to the specific population from which the sample was drawn. The lack of clinical follow-up does not allow for the assessment of the specific clinical impact of these variations on patient health. Also, the geographically homogeneous study population may represent a limitation for the applicability of the findings to the general population, as aortic arch anomalies might be more frequent in certain ethnic groups or areas. Further research involving larger, more diverse populations and utilizing a prospective design is necessary to provide a more accurate assessment of the prevalence and characteristics of aortic arch anomalies in the general population. Evaluating genetic and environmental factors that might contribute to the development of these anomalies could improve our understanding of why certain populations are more predisposed to these anomalies [105]. Concurrently evaluating the presence of cardiac malformations can help identify patients with higher risks for complications and allow for improved case management [106]. High-resolution CT is instrumental in intra-operative guiding for robotic surgery [107,108] and robotic-assisted corrections of vascular anomalies may be a viable future solution, especially in children or complex cases [109,110,111]. Multimodal studies on specific populations such as children may help to correctly assess the prevalence of aortic arch anomalies and to establish a base for a multidisciplinary approach to these variants.

## 6. Conclusions

Our paper included a large number of subjects in order to allow for the identification of a wide range of aortic arch variations which have a total prevalence in our study of under 1%. While the prevalence aligns with other studies worldwide, it is clear that several limitations need to be addressed in order to obtain a clearer and more realistic image of these variations in the general population, especially since numerous variations may be asymptomatic, leading to the underrepresentation of this category of patients.

While aortic arch anomalies are often discovered incidentally during imaging studies, a thorough understanding of these anatomical variations is crucial for effective patient management. We aimed to explore, depict, and discuss a selection of these conditions in order to provide a better comprehension of their complexity and imaging features.

Computed tomography is a non-invasive modality which allows for the correct detection and description of aortic arch anomalies based on the detailed visualization of anatomical relationships, the ability to perform advanced post-processing techniques (VR, MIP, MPR), and the potential to detect associated congenital anomalies or malformations.

## Figures and Tables

**Table 1:** Prevalence of aortic arch anomalies in our study.

Types of Aortic Arch Anomalies	Number (%)
Left aortic arch with the aberrant origin of the right subclavian artery	8 (44.44)
Emerging from the medial wall of the aortic arch	7 (38.89)
Emerging from the posterior wall of the aortic arch, associated with congenitally corrected transposition of great vessels and abnormal origin of the right vertebral artery from the ipsilateral common carotid artery	1 (5.55)
Right aortic arch	4 (22.22)
Type I	1 (5.55)
Type II	3 (17.64)
Ductus diverticulum	2 (11.11)
Double aortic arch	1 (5.55)
Aortic coarctation	1 (5.55)
Aortic pseudocoarctation	1 (5.55)
Patent ductus arteriosus	1 (5.55)

## Data Availability

The data that support the findings of this study are not openly available due to the reasons of sensitivity and are available from the corresponding author upon reasonable request.

## Abbreviations

AA	Ascending aorta
AO	Aorta
BT	Brachiocephalic trunk
CT	Computed tomography
cMPR	Curved multiplanar reformatting
DA	Descending aorta
LBT	Left brachiocephalic trunk
LCCA	Left common carotid artery
LSA	Left subclavian artery
LVA	Left vertebral artery
LV	Left ventricle
MPR	Multiplanar reformatting
PA	Pulmonary artery
RAA	Right aortic arch
RCCA	Right common carotid artery
RSA	Right subclavian artery
RV	Right ventricle
RVA	Right vertebral artery
SVC	Superior vena cava
VRT	Volume rendering technique
